# 4384 Factors Impacting Access to Gender Affirming Care for Gender Diverse Youth in the United States

**DOI:** 10.1017/cts.2020.268

**Published:** 2020-07-29

**Authors:** Kacie Kidd, Amber L. Hill, Gina M. Sequeira, Scott Rothenberger, Kristin N. Ray, Elizabeth Miller, Gerald T. Montano

**Affiliations:** 1 University of Pittsburgh

## Abstract

OBJECTIVES/GOALS: Access to pediatric subspecialty care varies by sociodemographic factors. Providers for gender diverse youth (GDY) are rare, and GDY face health disparities, stigma, and discrimination. We examined the association between GDY access to medical and mental health care and rurality, race, parental education, and other GDY-specific factors. METHODS/STUDY POPULATION: We surveyed parents of GDY (<18 years old) across the United States. Participants were recruited through online communities and listserves specific to parents of GDY. We determined associations between access to gender-specific medical or mental health providers and rurality, race, parental education, as well as other GDY-specific factors including age, time since telling their parent their gender identity, parent-adolescent communication, parent stress, and gender identity using chi-square or Fisher’s exact tests. We calculated adjusted odds ratios using logistic regression models. RESULTS/ANTICIPATED RESULTS: We surveyed 166 parents and caregivers from 31 states. The majority (73.2%) identified as white, 66.5% had earned a bachelor’s degree or higher, and 7.6% lived in a zip code designated rural by the Federal Office of Rural Health Policy. We found no evidence of association between reported GDY access to medical or mental health care and race, parental education, or rurality. We did find a significant univariate association between access to mental health care and feminine (either female or transfeminine/transfemale) gender identity (p = 0.033, OR 2.60, 95% CI 1.06 – 6.36). After controlling for parent-adolescent communication in a backwards elimination logistic regression model, it was no longer significant (p = 0.137, OR 2.05, 95% CI 0.80 – 5.25). DISCUSSION/SIGNIFICANCE OF IMPACT: Despite rurality, race, and parental education impacting access to pediatric subspecialty care, we failed to find these associations among GDY accessing gender care. There is a need to better understand structural and societal barriers to care for this population including the impact of stigma and discrimination.

